# COMAN: a web server for comprehensive metatranscriptomics analysis

**DOI:** 10.1186/s12864-016-2964-z

**Published:** 2016-08-11

**Authors:** Yueqiong Ni, Jun Li, Gianni Panagiotou

**Affiliations:** Systems Biology & Bioinformatics Group, School of Biological Sciences, The University of Hong Kong, Pokfulam Road, Hong Kong, Hong Kong

**Keywords:** Metatranscriptomics, Microbial RNA-Seq, Web servers, Microbial community, Computational biology

## Abstract

**Background:**

Microbiota-oriented studies based on metagenomic or metatranscriptomic sequencing have revolutionised our understanding on microbial ecology and the roles of both clinical and environmental microbes. The analysis of massive metatranscriptomic data requires extensive computational resources, a collection of bioinformatics tools and expertise in programming.

**Results:**

We developed COMAN (Comprehensive Metatranscriptomics Analysis), a web-based tool dedicated to automatically and comprehensively analysing metatranscriptomic data. COMAN pipeline includes quality control of raw reads, removal of reads derived from non-coding RNA, followed by functional annotation, comparative statistical analysis, pathway enrichment analysis, co-expression network analysis and high-quality visualisation. The essential data generated by COMAN are also provided in tabular format for additional analysis and integration with other software. The web server has an easy-to-use interface and detailed instructions, and is freely available at http://sbb.hku.hk/COMAN/

**Conclusions:**

COMAN is an integrated web server dedicated to comprehensive functional analysis of metatranscriptomic data, translating massive amount of reads to data tables and high-standard figures. It is expected to facilitate the researchers with less expertise in bioinformatics in answering microbiota-related biological questions and to increase the accessibility and interpretation of microbiota RNA-Seq data.

**Electronic supplementary material:**

The online version of this article (doi:10.1186/s12864-016-2964-z) contains supplementary material, which is available to authorized users.

## Background

In the era of next-generation sequencing (NGS), microbiome-oriented studies have recently been the “hot spot” and have deepened our understanding on the crucial roles of both clinical and environmental microbes. The field of shotgun metagenomics investigating the genetic potential of microbiota has blossomed out, shedding light on microbial ecology, evolution and disease biology. Metatranscriptomics investigates the totality of the expressed genes in a microbial community under particular conditions. This RNA-based profiling of microbial community structure and function can reflect the actual expressed activity of involved microbiota unseen by metagenomics, and has been applied mainly to environmental microbiota during early stage [1, 2]. The subsequent application of metatranscriptomics to human gut microbiota [3, 4] has revealed actively transcribed core modules, inter-subject and temporal gene expression variations. More importantly, metatranscriptomics can also unveil the microbial responses to altered environmental conditions (e.g. disease versus health) or other external stimuli. Dietary [5] and xenobiotic treatments [6] have been found to alter significantly gut microbial gene expression profiles, while health- and disease-associated oral microbiota communities displayed defined metabolic differences [7].

The use of NGS in metatranscriptomics generates large datasets with high degree of complexity, which needs to be analysed effectively to translate the non-interpretable raw sequencing reads to biological insights, in the format of data tables and figures. Although a few relevant methods or pipelines for processing RNA-Seq data have been proposed [8–10], the whole analysis process for such high-throughput data typically involves many individual steps, the installation and execution of a wide range of software tools, extensive computational resources and expertise in programming and NGS bioinformatics data analysis. Particularly, Leimena et al. [9] described in detail a pipeline, or guidelines, for analysing metatranscriptomic Illumina RNA-seq data, but did not implement it as a software tool or web-based server. MetaTrans [11] and SAMSA [12] are the tools recently developed specifically for metatranscriptomics, but they require proper local setup on a powerful computer cluster or offer limited functional analysis. Therefore, even though metatranscriptomic data are now routinely generated, it is challenging for wet-lab researchers to analyse them and generate biological relevant information. Despite that web-based servers such as MG-RAST [13] and EBI Metagenomics [14] can be also adopted for metatranscriptomics, they were originally designed to process and annotate metagenomic data with limited functionality in uncovering and elucidating the active microbial functions and responses to external stimuli hidden in metatranscriptomics.

Here we present COMAN, a web-based application for functional characterisation and comprehensive analysis of high-throughput metatranscriptomic data. COMAN processes uploaded raw reads automatically to ultimately achieve functional assignments, which are then used to perform comparative statistical analysis, pathway enrichment and co-expression network analysis, to relate taxonomy with functional variations and to visualize the results. With an easy-to-use interface and extensive instructions, COMAN can be run by experimentalists without programming experience and without the hassle of changing tools or working environments for answering their biologically relevant questions. However, since the essential data are also provided in tabular format, users with bioinformatics expertise may perform additional analysis and integration with other software.

## Implementation

COMAN accepts as input the Illumina paired-end sequencing reads in FASTQ format and a metadata file specifying the sample conditions for comparative statistical analysis. A sample input dataset is offered to guide users on data formatting requirements (by clicking the “SampleData” button in the homepage). It is optional to upload a file containing the metagenomic taxonomic abundance profiles, which will only be used for “transcription activation analysis” (as described below). All uploaded data and results generated by COMAN are restricted to the user who initiated the job. A comprehensive guidance on COMAN usage can be found in the “Instructions” section of the server homepage.

**Fig. 1:**
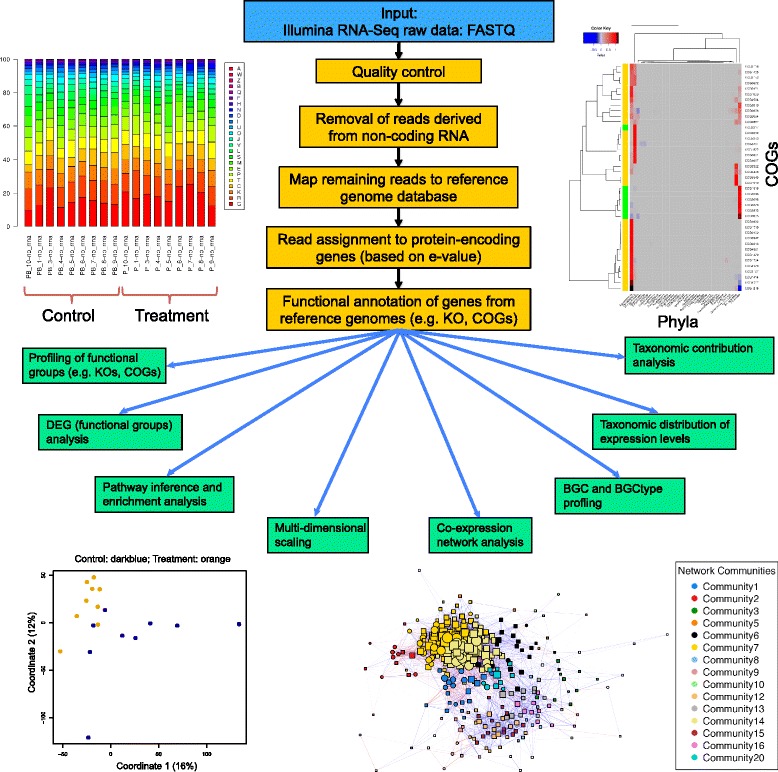
The metatranscriptome analysis pipeline in COMAN. The example output figures from the analysis of a gut microbiome dataset are shown. Upper left: functional profiling; upper right: taxonomic contribution analysis (complete linkage method for clustering algorithm and Euclidean distance for dissimilarity metric); lower left: multi-dimensional scaling to illustrate sample clustering; lower right: co-expression network analysis with different inferred communities (for clarity purpose, the communities with fewer than 3 elements are merged together and not highlighted here)

The whole COMAN metatranscriptome data analysis pipeline (Fig. 1) mainly involves quality control, removal of reads derived from non-coding RNAs, functional annotation, comparative statistical analysis, taxonomy-associated functional analysis and co-expression network analysis. Details on key steps are shown below:

### Quality control and removal of non-coding RNA

The uploaded NGS reads are subject to an initial quality control step to remove the adapter regions and low quality reads following a previously described approach [15]. Afterwards, all the QC-passed reads are mapped, using BLASTN, to an in-house non-coding RNA database (see Results for details and evaluation) to filter out the reads derived from non-coding RNAs, including ribosomal RNA and tRNA. The reads with best BLAST hits at e-value < 10^−5^ are excluded from downstream analysis.

### Mapping to reference genomes

In this step, all the high quality reads after depletion of non-coding RNA are further mapped to a reference genome database at 1e-5 cutoff, which includes more than 2700 NCBI complete microbial genomes (accessed at ftp://ftp.ncbi.nlm.nih.gov/genomes/archive/old_refseq/Bacteria/all.faa.tar.gz). We used a much faster and highly sensitive tool named DIAMOND [16] within the COMAN pipeline, since using BLASTX to perform this task is too time-consuming and not practically feasible, especially for a web-based application.

### Functional annotation of genes and reads

Functional annotations of those reference genomes have been pre-prepared at the COMAN backend. This includes commonly used annotation systems: Clusters of Orthologous Groups (COG) [17] and KEGG Ortholog groups (KO) [18]. The annotation to COG was conducted using RPS-BLAST against the CDD database (v.3.10) at 1e-5 cutoff, whereas the KO annotation was through the combinatorial use of DIAMOND and KOBAS 2.0 *annotate* program [19]. In addition, we used PRIAM (with default parameters) [20] to annotate the genes to enzymes (Enzyme Commission numbers) (ECs) that are further used to achieve the profiling of MetaCyc pathways [21].

### Comparative statistical analysis

Based on the mapping of reads to reference genomes and the functional annotation results, COMAN performs functional profiling and calculates the relative abundance for each functional group and enzyme, as well as for a higher hierarchy level in the annotation system. This high-level profiling includes COG categories, KEGG pathways, KEGG pathway classes, KEGG modules, and various levels of MetaCyc pathways.

The clustering of all samples using multidimensional scaling (MDS) is incorporated within the pipeline. Once the functional profiling based on COG and KO is completed, the metadata file will be used to conduct differential expression (DE) analysis (Wilcoxon rank sum test, with the default cutoff of FDR-corrected *p-value* being 0.10), in order to characterise the potential functional changes between two different conditions.

Researchers are often highly interested in the associations between pathway variations and biological phenotypes observed. To facilitate such process, we integrate pathway inference and analysis based on KEGG and MetaCyc into the COMAN pipeline. Despite certain overlap and differences [22], they are both commonly used pathway systems during genomic analysis and metabolic reconstruction, and users of COMAN will have access to results derived from both databases. Most importantly, COMAN uses MinPath [23] to infer the pathways represented in the submitted microbial communities, based on functional annotations of genes and the relationship between pathways and functional groups (KO for KEGG; EC for MetaCyc). Compared with the naive one-hit-match mapping approach that may inflate the number of inferred pathways leading to overestimation of functional diversity, MinPath eliminates some spurious pathways and achieves a more trustworthy inference of biological pathways present in the samples [23].

Afterwards, COMAN incorporates GAGE [24] to infer the significantly enriched or depleted pathways when comparing two conditions. Users may apply a suitable threshold of FDR-corrected *p-values* to identify such pathways that display coordinated differential expression over the whole pathway and that can be associated with the biological phenotypes. This pathway enrichment analysis is applied to KEGG pathways, KEGG modules and MetaCyc pathways.

Functional profiling, differential expression and pathway enrichment analysis elucidate the functional states and dynamic changes or responses of the involved microbiota community. It is also useful to relate the observed functional variations with particular taxa [25]. For this purpose, COMAN performs a taxonomic contribution analysis to identify which microbial phylum is mostly responsible for community-level expression variation, for each of the most varied functional groups (Fig. 1). These “most varied functional groups” refer to either significantly differentially expressed ones based on user-specified FDR-corrected *p-value*, or the ones with highest fold-change (up- 50 and down-regulated 50 groups) when the former is not available. Last but not least, COMAN incorporates a taxonomic distribution analysis, where the expression distributions of each functional group among different phyla at both conditions are calculated.

### Transcription activation analysis

If the user has the taxonomic abundance profiles across all samples generated by metagenomic sequencing and analysis, this file (phylum-level) can be uploaded to COMAN at the data uploading process. Afterwards, COMAN normalises the gene expressions using the taxonomic abundances, followed by comparison and visualisation of the normalised expression levels in two conditions. This further elucidates whether the observed expression variations of certain genes are derived from varied transcription levels (transcriptional activation or repression) or simply caused by bacterial taxonomic composition differences.

### Co-expression network analysis

In co-expression network, genes having similar or related functions tend to possess similar expression profiles and thus tend to be clustered together [26, 27]. COMAN calculates the pairwise correlations (Spear correlation) for the “most varied functional groups” aforementioned, based on their expression profiles among different samples in one condition, and generates a co-expression network accordingly. Afterwards, the random walk algorithm is used to find densely connected sub-networks, or communities within the network (Fig. 1). The elements within the same community represent concordant behaviours, such as similar responses to a particular stimulus, and thus the similar or closely related functions.

The results from COMAN co-expression analysis include TAB-delimited data files presenting the involved functional groups and their topological properties and belonged communities, as well as high-quality figures for network visualisation with deduced communities (Fig. 1). Moreover, since such network typically contains extensive information, a web-based interactive network is provided in the result page for deeper inspection. For clarity purposes, all detected communities with fewer than 3 elements are merged into one single residual module. Users may also investigate the “hub nodes” in the resulting network, which represent genes or functional groups with extremely high connectivity.

Despite that only the “most varied functional groups” are involved in COMAN, users may use the abundance profiles for all functional groups or any subset of interest (COGs, KOs, or ECs) to construct a global co-expression network and perform network topological analysis. Note that to perform a meaningful co-expression network analysis using COMAN, the minimal number of samples within one condition has to be greater than four. However, we recommend the sample size to be larger than eight in order to generate findings of more biological relevance with lower false positives.

### Profiling of Biosynthetic Gene Clusters for analysis of microbial secondary metabolites

Biosynthetic gene clusters (BGCs) are physically clustered gene sets responsible for the synthesis of microbial secondary metabolites, whose importance and widespread distribution in the human microbiome have been demonstrated before [28]. Despite the greater understanding of different microbiota in varying environments, there is still paucity of characterised metabolites that are synthesized by the microbial community and that may contribute to the differential phenotypes [15]. Here we plugin antiSMASH 3.0 [29] for BGC annotation into the COMAN pipeline, to facilitate the characterisation and comparison of secondary metabolite biosynthesis among different conditions. Using our pre-prepared identification of BGCs from the NCBI reference genomes, the BGCs and their abundances in each submitted sample can be calculated following similar rules as Donia et al [28]: 50 % of genes (after excluding non-biosynthetic ones) in each BGC need to be covered by reads; the abundance score of this identified BGC is defined by taking the average abundance of these genes. Further, the abundances of BGCs producing different types of secondary metabolites (BGC types, as defined by antiSMASH) are calculated, followed by a comparative statistical analysis (Wilcoxon rank sum test) to identify differentially abundant BGC types. This reveals the active production capability of different types of secondary metabolites by the involved microbiota. A bar chart illustrating the relative proportion of different types is provided as well. Note that this BGC-related analysis module in COMAN reflects the transcriptionally active BGCs in the microbial community.

### Results visualisation and access

COMAN produces a collection of easy-to-interpret figures upon job termination. The example output of a complete dataset derived from a human gut microbiome project is available on the server (the “Example Data” section of the server homepage). The essential data in tabular format (“TAB” delimited file) are provided, therefore the user may use them to perform additional analyses, which include the integration with other tools as well as more sophisticated analyses for advanced users. For relatively basic figures such as bar charts, they can make custom modifications of figures using their preferred programs. For co-expression network visualisation, users may choose CytoScape with a graphical user interface or use the R script provided (Additional file 1) to apply different filtering and layout parameters to the network. Moreover, COMAN keeps the raw sequence mapping files in BLAST m8 format that includes the details of sequence alignment (e.g. identity, alignment position, e-value). A detailed documentation called “README.txt” describing each output file is included for every completed job to help users interpret the results.

All results generated by COMAN are compressed to simplify data download. Since the sequence mapping results are high data volume and take considerably more time to download, COMAN separates such files with those generated by functional analysis for the convenience of users.

## Results and discussion

### Construction and evaluation of non-coding RNA database

The removal of reads derived from non-coding RNA can avoid the potential bias during downstream analysis, but would normally take a few days for all sequenced samples and even up to one month for larger datasets with deep sequencing. Since the purpose here is only to identify and remove those reads, rather than to determine their taxonomic origins, there would be great extent of redundancy within the non-coding RNA database such as SILVA [30]. To reduce the computational burden and accelerate our pipeline, we constructed and evaluated an in-house non-coding RNA database, which is a random 10 % subset of the combination of 1) NCBI bacterial reference genomes non-coding RNAs (accessed at ftp://ftp.ncbi.nlm.nih.gov/genomes/archive/old_refseq/Bacteria/all.frn.tar.gz); and (2) eukaryotic ribosomal DNA (both large and small subunits) within SILVA (See Additional file 2 for details in construction and evaluation of the database).

**Fig. 2:**
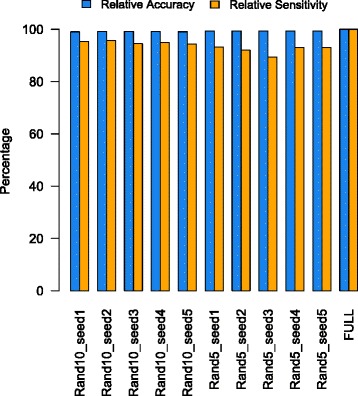
Performance evaluation of subsets of the combined database used in COMAN. The combined database was constructed by merging the NCBI bacterial reference genomes non-coding RNAs with eukaryotic ribosomal DNA (both large and small subunits) deposited in the SILVA database. Different subsets of random 10 % and 5 % of the full combined database (indicated by x-axis) were taken and their performance was compared to the BLASTN mapping results from using the full version. For each subset, the “Relative Accuracy” is defined as the number of commonly identified reads between the subset and the full database, divided by the total number of reads identified only by using the subset for mapping. In comparison, the “Relative Sensitivity” is defined as the number of commonly identified reads between the subset and the full database, divided by the total number of reads identified by using the full database

We compared the performance of different random 10 % and 5 % subsets (5 % NCBI + 5 % SILVA), as well as the full combined database without taking subsets. It can be seen from Fig. 2 that while the 10 % subsets showed rather high accuracy, sensitivity and stability, the performance of random 5 % subsets was not very stable, with one of them having sensitivity even below 90 %. Being much less redundant, the final 10 % combined database reduces the mapping time remarkably (~6 folds) while remaining nearly the same sensitivity as compared to the full database. Using this in-house non-coding RNA database, the reads with best BLAST hits at e-value < 10^−5^ are excluded from downstream analysis.

### Case study

For illustration, we performed an analysis of a complete dataset from a human gut microbiota project [5] and the results preview can be accessed via the “Example Data” page. The MDS results generated by COMAN (Additional file 3) shows that samples from two different conditions (“control diet” and “plant-based diet”) separated clearly, indicating the functional alterations of human gut microbiome by plant-based diet. The subsequent taxonomic contribution analysis suggests that Firmicutes, Proteobacteria, Actinobacteria and Bacteroidetes were the main contributors of community-level variations for the most varied functional groups. Moreover, Firmicutes was found to contribute predominately to the up-regulated KEGG orthologous groups. Similar analysis with more focused discussion on the results has been performed in our previous attempt to understand the diet-gut metagenome interactions at a molecular level [25].

### Computational time and throughput

After optimising the sequence-mapping step, the whole COMAN analysis should be completed within a few days (typically 1-2 days). Using our example dataset that includes 18 samples with an average of around 22M reads per sample, the time elapsed was approximately 2.5 days. In relation to the functional analysis part, it was completed in less than 10 min. COMAN has the capacity to handle multiple jobs simultaneously and the time needed depends on the sequencing depth, the number of samples and the server workload. Users should expect that running 5 jobs in parallel with each job containing 10 samples with ~20M reads will require approximately one week to be completed. The users may check the results at a later time with the provided job ID, bookmarked pages or get notified by email upon job termination (given that an email address is provided).

### Other highlights

By using a large amount of complete microbial genomes, COMAN is able to perform functional annotation for more than one system, including the widely accepted KEGG, COG and MetaCyc, as well as a more specialised annotation for secondary metabolite biosynthesis - namely biosynthetic gene clusters (BGCs). Therefore, in addition to the comparative analysis aimed to shed lights on the links between functional variations and biological phenotypes, COMAN also serves as a versatile functional annotation tool that provides the fundamental data tables essential for in-depth analysis. More experienced users may use such results generated by COMAN with multiple functional annotation and pathway systems, directly or with slight data rearrangements, as the input of other specialised NGS data analysis programs such as LefSe [31].

## Conclusion

Here, we developed COMAN (Comprehensive Metatranscriptomics Analysis), which serves as a platform to translate the non-interpretable raw sequencing reads to data tables and high-standard figures that can be easily handled and further analysed. Although related theoretical methods or tools have been reported before, COMAN is an integrated web server dedicated to comprehensive functional analysis of metatranscriptomic data. It is easy-to-use and relatively fast, with extensive instructions on input data format, server navigation and results interpretation. The output includes high-quality figures in both PNG and PDF formats, as well as the essential data in tabular format. Therefore, COMAN is expected to facilitate the researchers, who may lack the expertise or computational resources, to analyse microbiota RNA-Seq data towards tackling their biological questions of interest. Advanced users with bioinformatics expertise may perform additional analysis and integration with other software by utilising the essential tabular data COMAN generates. With a wide range of target audience including microbiologists, environmental biologists and clinicians, we believe COMAN will benefit the community remarkably in revealing the importance of both environmental and clinical microbiota. Considering the availability of powerful computational resources, the current pipeline is only run on our server in a web-based manner. However, making COMAN pipeline as a separate software package that can be setup locally and run in a fully customised way is in our plans. The COMAN server will be actively and continuously updated to incorporate more annotation systems and analysis modules (e.g. taxonomy-related analysis at high-resolution) in the future.

### Availability and requirements

- Project name: COMAN
- Project home page: http://sbb.hku.hk/COMAN
- Operating system(s): Platform-independent
- Programming language: Python, R
- License: This server is free to all users without login requirement

## Abbreviations

BGC, Biosynthetic gene clusters; COG, Clusters of Orthologous Groups; DE, Differential Expression; EC, Enzyme Commission numbers; KO, KEGG Ortholog groups

## Additional files

Additional file 1: The R script to visualise the co-expression network. The input file is generated by COMAN co-expression analysis. (R 8 kb) Additional file 2: Supplementary results detailing the process of constructing and evaluating the non-coding RNA database used in COMAN. (DOC 345 kb) Additional file 3: Clustering of all samples from the example data using Multidimensional Scaling based on the abundances of all COG groups. Dark blue: control or baseline diet; orange: after plant-based diet. (PDF 5 kb)
