# Supplementary material for: COMAN: a web server for comprehensive metatranscriptomics analysis
Source: BMC Genomics. 2016 Aug 11;17:622. doi: 10.1186/s12864-016-2964-z (PMC4982211; doi:10.1186/s12864-016-2964-z)
Supplement: Additional file 2: — Supplementary results detailing the process of constructing and evaluating the non-coding RNA database used in COMAN. (DOC 345 kb) [file 12864_2016_2964_MOESM2_ESM.doc]

**(Supplementary Materials)**

**COMAN: a web server for COmprehensive Metatranscriptomics Analysis**

Yueqiong Ni^1^, Jun Li^1^, Gianni Panagiotou^1,*^

^1^Systems Biology & Bioinformatics Group, School of Biological Sciences, The University of Hong Kong, Pokfulam Road, Hong Kong

* To whom correspondence should be addressed. E-mail: gipa@hku.hk Phone: (852) 2299-0349

**Construction of non-coding RNA database**

***Random 10% subset of non-coding RNAs from more than 2700 NCBI reference genomes (including bacteria and archaeal)***

First, we used all the non-coding RNAs from NCBI complete microbial genomes (including bacteria and archaea, accessed at <ftp://ftp.ncbi.nlm.nih.gov/genomes/archive/old_refseq/Bacteria/all.frn.tar.gz>) as the full database for searching the reads derived from non-coding RNAs. A series of subsets were then randomly extracted from the full database. Then the same example data file with 1M query reads (randomly selected from a full sample) was mapped using BLASTN against those subsets and the full database. The reads with best BLAST hits at e-value < 10^-5^ were regarded as the ones derived from non-coding RNAs. For each database subset, we summarised the number of reads identified by this subset, as well as the reads commonly identified by both the subset and the full database. We then calculated the “Relative Accuracy” and “Relative Sensitivity” and illustrated them in Supplementary Figure 1. For each subset, the “Relative Accuracy” is defined as the number of overlapped reads between the subset and the full database, divided by the total number of reads identified by using the subset for mapping. In comparison, the “Relative Sensitivity” is defined as the number of overlapped reads between the subset and the full database, divided by the total number of reads identified by using the full database.

It can been observed that as the percentage of the random subset decreases, the accuracy for identifying non-coding RNAs remained highly stable at the level of higher than 98%. However, the sensitivity decreased gradually especially when small subsets were used such as 0.5% and 1%. Therefore, we used the random 10% subset as the reduced database constructed from NCBI microbial genomes, due to nearly the same performance as the full database (Relative Accuracy: 98.5%; Relative Sensitivity: 97%).


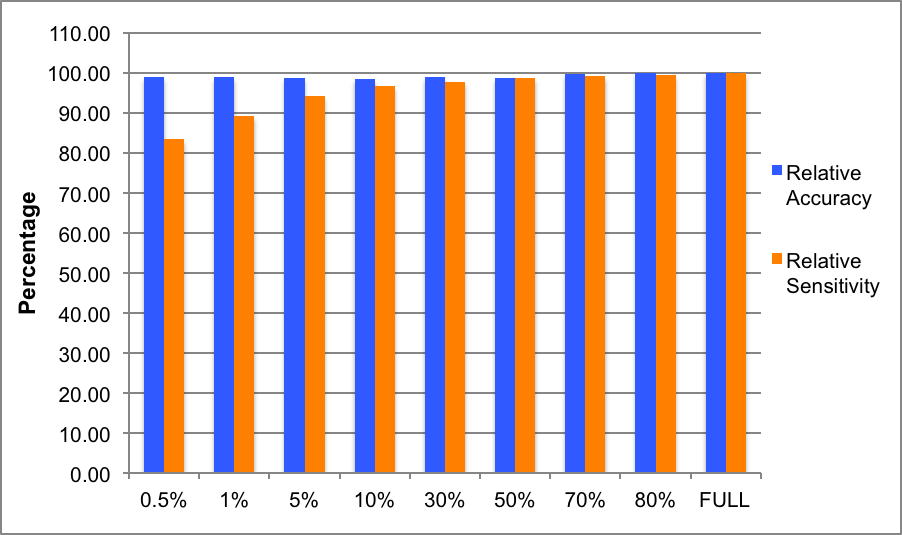


Supplementary Figure 1: Evaluation of the redundancy of the non-coding RNA database constructed from NCBI complete microbial genomes. A range of subsets of the full database were taken and their performance was compared to the full version by performing a BLASTN mapping of a sample with 1M randomly-selected query reads.

***Random 10% subset of SILVA Eukaryotic LSU and SSU ribosomal DNA***

Then we also evaluated the redundancy of non-coding RNA in a commonly used database, the SILVA database . We took a series of subsets (from 10% to 80%) of SILVA database (version SILVA 123) including both SSU (small subunit ribosomal RNA) and LSU (large subunit ribosomal RNA), performed the same mapping and summarised with the same threshold as evaluating the NCBI reference genomes. The relative accuracy and sensitivity were calculated and presented in Supplementary Figure 2.

Again, both the accuracy and sensitivity remained highly stable for all subsets taken. The random 10% subset achieved an accuracy of 99.4% and a sensitivity of 97%, demonstrating its suitability for being a reduced subset of the SILVA database.


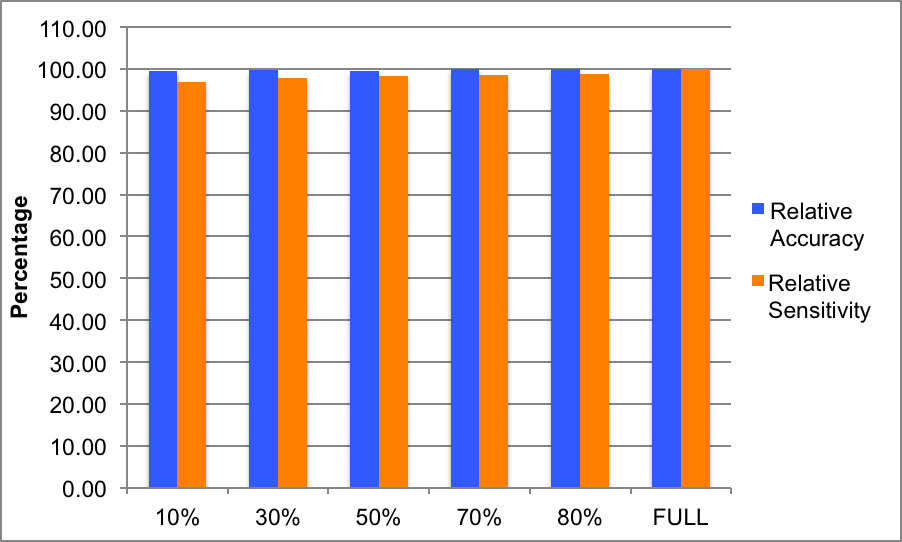


Supplementary Figure 2: Evaluation of the redundancy of the SILVA ribosomal DNA database. A range of subsets of the full database were taken and their performance was compared to the full version by performing a BLASTN mapping of a sample with 1M randomly-selected query reads.

Afterwards, instead of comparing each SILVA subset with the full database, we compared their performance with the corresponding subsets taken from the non-coding RNA of NCBI reference genomes aforementioned.


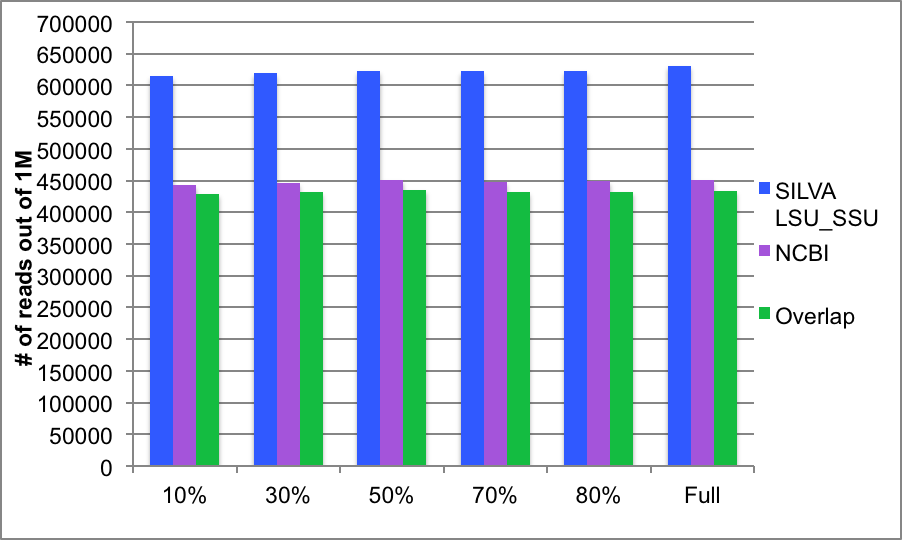


Supplementary Figure 3: Comparison of the performance of the SILVA database and the database constructed from NCBI microbial genomes. The query data includes 1M randomly selected reads from a sample.

We found that the overlap between SILVA and NCBI was high and stable, and a 10% subset could give rise to nearly the same results as the full database. There were reads that were only identified by using SILVA (Supplementary Figure 3), which should represent the non-coding RNA derived from eukaryotes, since the ribosomal RNAs deposited in SILVA include both prokaryotic and eukaryotic organisms. We then retrieved only the hits mapped to Bacteria and Archaea and did the comparison again. The results for identification of non-coding RNAs using SILVA and NCBI genomes were found to be highly consistent (Supplementary Figure 4). The slightly higher number of reads for NCBI genomes should mainly originate from tRNA and other types of non-coding RNAs.


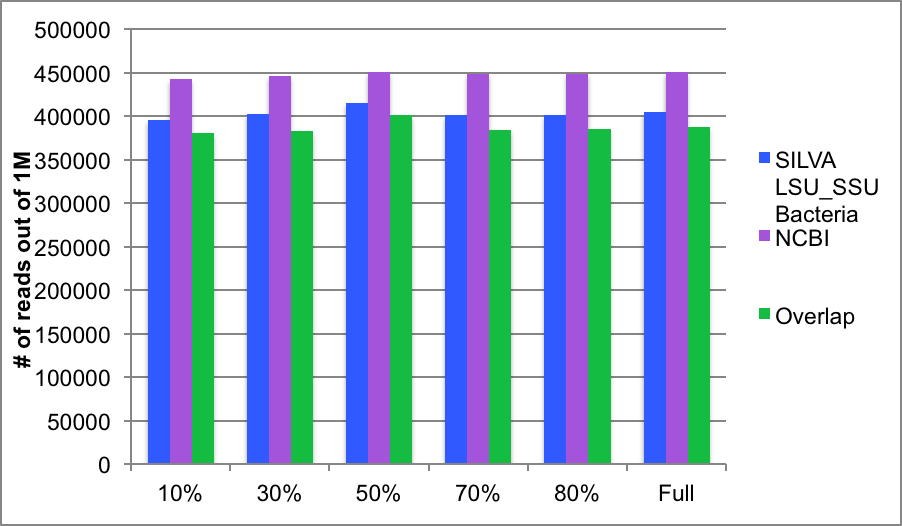


Supplementary Figure 4: Comparison of the performance of the SILVA database and and the database constructed from NCBI microbial genomes in identifying bacteria-derived non-coding RNAs. The query data includes 1M randomly selected reads from a sample.

***The combined database for non-coding RNA removal in COMAN***

Based on above evaluation, we combined the NCBI bacterial reference genome non-coding RNAs and the eukaryotic ribosomal DNA (both large and small subunits) within the SILVA database. For the combined full database, different random 10% (10% NCBI + 10% SILVA) and 5% subsets (5% NCBI + 5% SILVA) were extracted and evaluated for their performance compared with full database. It can be seen from Figure 2 that while the 10% subsets showed rather high accuracy, sensitivity and stability, the performance of random 5% subsets was not very stable, with one of them having sensitivity even below 90%.


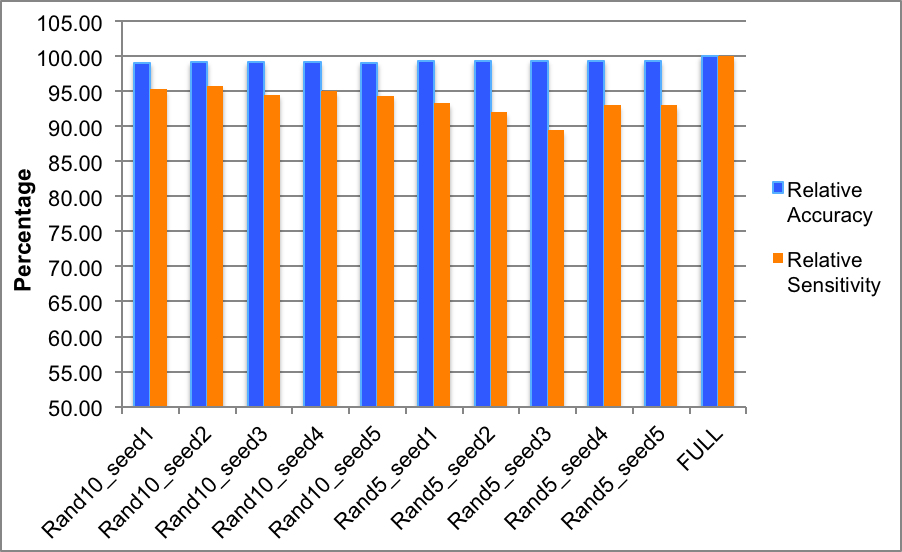


Supplementary Figure 5: Evaluation of the performance of the subsets of combined database used in COMAN. The combined database was constructed by merging the NCBI bacterial reference genomes non-coding RNAs with eukaryotic ribosomal DNA (both large and small subunits) deposited in the SILVA database. Different subsets of random 10% and 5% of the full combined database were taken and their performance was compared to the BLASTN mapping results using the full version.

Therefore, taken all into account, we used as the final non-coding RNA database the combination of 1) random 10% subset of all non-coding RNAs from NCBI bacterial reference genomes; and 2) random 10% subset of those eukaryotic ribosomal DNA within the SILVA database.

**References**

**Quast, C.*, et al.* (2013) The SILVA ribosomal RNA gene database project: improved data processing and web-based tools, *Nucleic Acids Res*, 41, D590-596.**
