# Supplementary figures and images for: COMAN: a web server for comprehensive metatranscriptomics analysis
Source: BMC Genomics. 2016 Aug 11;17:622. doi: 10.1186/s12864-016-2964-z (PMC4982211; doi:10.1186/s12864-016-2964-z)

**Control: darkblue; Treatment: orange**

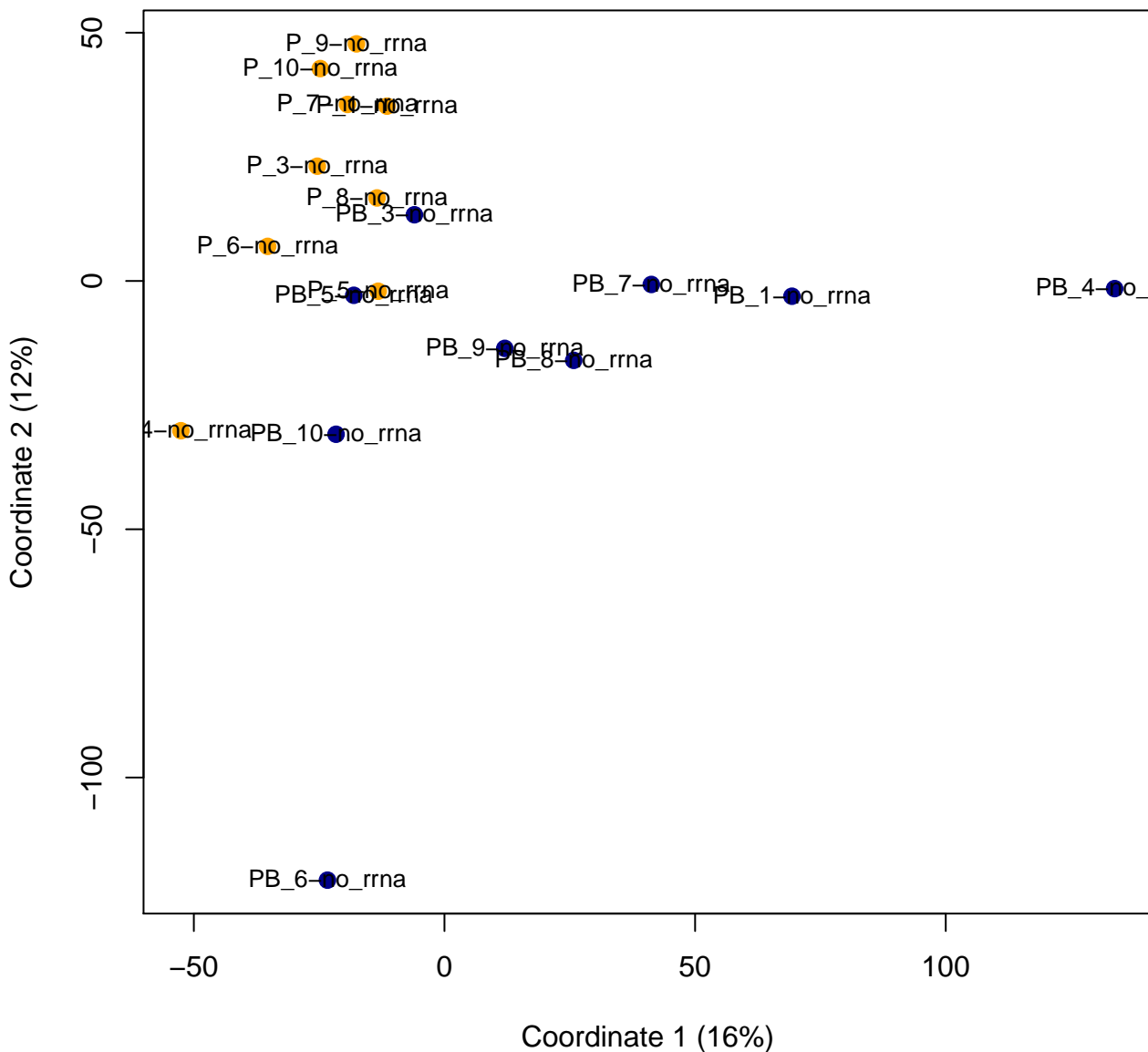

Supplement: Additional file 3: — Clustering of all samples from the example data using Multidimensional Scaling based on the abundances of all COG groups. Dark blue: control or baseline diet; orange: after plant-based diet. (PDF 5 kb) [file 12864_2016_2964_MOESM3_ESM.pdf]
